# Digital clinical placements: Student perspectives and preparedness for placements

**DOI:** 10.1111/tct.13558

**Published:** 2023-01-04

**Authors:** Natasha Houghton, Lucy Williams, Ana Baptista, Viral Thakerar, Aynkaran Dharmarajah

**Affiliations:** ^1^ Centre for Engagement and Simulation Science Imperial College London London UK; ^2^ Faculty of Medicine Imperial College London London UK; ^3^ Medical Education Research Unit, Faculty of Medicine Imperial College London London UK

## Abstract

**Background:**

In May 2020, first‐year students at Imperial College School of Medicine attended a ‘digital hospital placement’. Occurring in the early months of the COVID‐19 pandemic, this replaced their first planned hospital placement. The authors analysed student experiences to understand how a digital hospital placement impacted self‐perceived clinical and professional development and whether it improved preparedness for face‐to‐face hospital placements.

**Methods:**

Three hundred ten students participated in this week‐long digital placement, which integrated clinical skills, communication and professional behaviour domains. It aimed to prepare students for safe participation in clinical environments. Resources included self‐directed and peer learning, virtual simulations (Oxford Medical Simulation) and staff‐led debriefing. Surveys were administered after the digital placement and after students' first face‐to‐face placement to collect quantitative and qualitative data. A reflexive thematic analysis was conducted.

**Results:**

Eighty‐three and twenty‐nine students completed the postdigital and post‐face‐to‐face placement evaluation respectively. Quantitative results indicated a high self‐rated achievement of learning objectives and enthusiasm for digital placements; 83% of respondents supported digital simulations as part of regular medical education. Qualitative analysis identified three superordinate themes: (1) domain integration in digital placements helped students feel better prepared; (2) digital experiential learning is ideally suited to early clinical learning; and (3) virtual placements are a compliment, not an alternative, to face‐to‐face placements.

**Conclusion:**

Digital placements are a promising means of supporting the challenging transition from classroom learner to clinical learner. They provide a feasible and scalable option for building student confidence and improving preparedness.

## 1 INTRODUCTION

First‐year students at Imperial College School of Medicine (ICSM) were scheduled to attend their first hospital placement in May 2020. The arrival of COVID‐19 forced educators to rapidly transition to remote online teaching, posing a major challenge.^1^, ^2^ Although a wealth of literature documents this shift to online learning during the COVID‐19 pandemic,^3^ much of this is limited to ‘emergency remote teaching’ in a single domain, often lacks grounding in educational theory, is based on small sample sizes and evaluated low levels of learning.^3^ Rigorous evaluation of COVID‐19 era innovations is necessary not only to prepare for possible future disruptions but also to determine what would be valuable in ‘routine medical education’ moving forwards.^4^ This study investigates the student perceptions of a 1‐week digital hospital placement attended by 310 ICSM year 1 students. Our research questions were as follows:

1. Do students perceive digital placements to be useful preparation for hospital placements?
2. To what extent do digital placements impact self‐perceived clinical and professional development?

Our results highlight the potential role of digital placements as a long‐term adjuvant to hospital placements to aid preparedness for early clinical experiences.

### 1.1 Digital placement development

**FIGURE 1:**
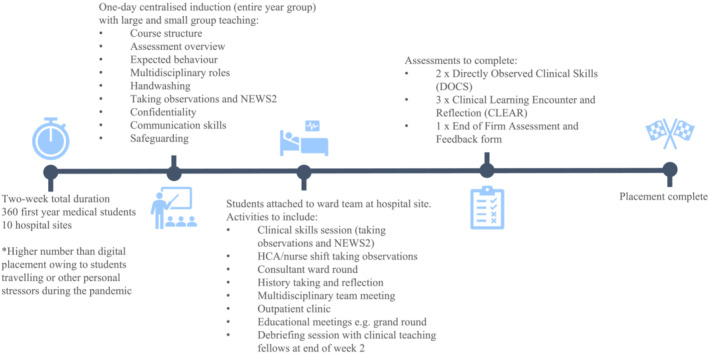
Original placement design

The original design pre‐pandemic involved a 1‐day centralised induction followed by a 2‐week structured hospital placement in which students were assigned to a ward to complete various clinical activities and assessments (as detailed in Figure 1). Although our initial design had sought to prepare students for their placement through the delivery of a centralised induction, this was limited to 1 day. Existing evidence shows students do not feel adequately prepared for clinical placements^5^ and the transition from classroom to clinic in pre‐clinical years is particularly challenging.^6^ In designing the digital placement, our aim was to increase preparedness for face‐to‐face hospital placements and do so using an integrated approach to curriculum design. Our institution employs a spiral curriculum, combining both horizontal and vertical domains, which allows concepts to evolve across intersecting subject matter over time.^7^ This requires committed cooperation across different departments.^8^ Eight academic teaching staff from three domains (clinical skills, professional values and behaviours and communication skills) collaborated to design the digital placement to ensure the integration of these three elements. Of note, the clinical skills domain also included teaching on the basic science and knowledge underpinning required skills. For example, ‘skills learning’ when taking observations and vital signs included teaching on the physiology of haemodynamic regulation. We used a combination of bespoke resources made by faculty (instructional videos and video diaries from patients and doctors, discussion guides and pre‐reading) and pre‐existing interactive material from external organisations (Oxford Medical Simulations^9^ and Royal College of Physicians NEWS2 e‐Learning tool^10^). Structure, activities and learning outcomes are summarised in Table 1.

**TABLE 1:** Digital placement learning activities

Domain	Intended learning outcomes	Activities	Resources	Learning theory
Clinical Skills and Acute Medicine	Patient safety: the importance and technique for handwashing Procedural skills: how to safely and correctly obtain and document basic observations How to calculate a NEWS2 from basic observations and escalate appropriately Observations: describe at least one scenario where each observation is abnormal	Two self‐directed tutorials: Calculating NEWS2 Handwashing Two OMS simulations Asthma Pulmonary Embolism Q&A with faculty Live webinar debrief with faculty	Pre‐reading material, interactive puzzles and quizzes Pre‐recorded video demonstration by staff NEW2 Royal College of Physicians NHS eLearning tool Oxford Medical Simulation	Kolb's experiential learning cycle Knowles' theory of andragogy
Time for activities: 8‐h plus 2‐h webinar with faculty				
Professional Values and Behaviours	Biopsychosocial model: Describe the biopsychosocial model and the need to take account of the lived experience of illness Person‐centred care: Describe key principles of person‐centred care, and how these relate to exploring a person's understanding, values and preferences, including shared decision‐making, social prescribing and health literacy Apply the principles of reflection on clinical interactions Understand the role of different members of the health service Understand the role of medical students within the health service	Two self‐directed tutorials completed in pairs: Experience of illness Navigating the system Q&A with faculty Live webinar debrief with faculty	Pre‐reading material, video footage of interviews with patients (Healthwatch) and doctors Discussions guides to analyse videos (in pairs) Reflective portfolio	Kolb's experiential learning cycle Social constructivism
Time for activities: 4‐h plus 2‐h webinar with faculty				
Clinical Comms	Describe how good communication underpins the delivery of quality healthcare in the acute setting Describe the effect of communication on clinician–patient interaction Describe the effect of communication between health professionals within teams	Two self‐directed tutorials completed in pairs: Handover On the ward round Q&A with faculty (online) Live webinar debrief	Video examples of optimal and sub‐optimal consultations Video examples of simulated ward rounds Discussion guides to analyse videos Pair activities to practise handovers	Kolb's experiential learning cycle Social constructivism
Time for activities: 4‐h plus 2‐h webinar with faculty

Our aim was to increase preparedness for face‐to‐face hospital placements and do so using an integrated approach to curriculum design.

**FIGURE 2:**
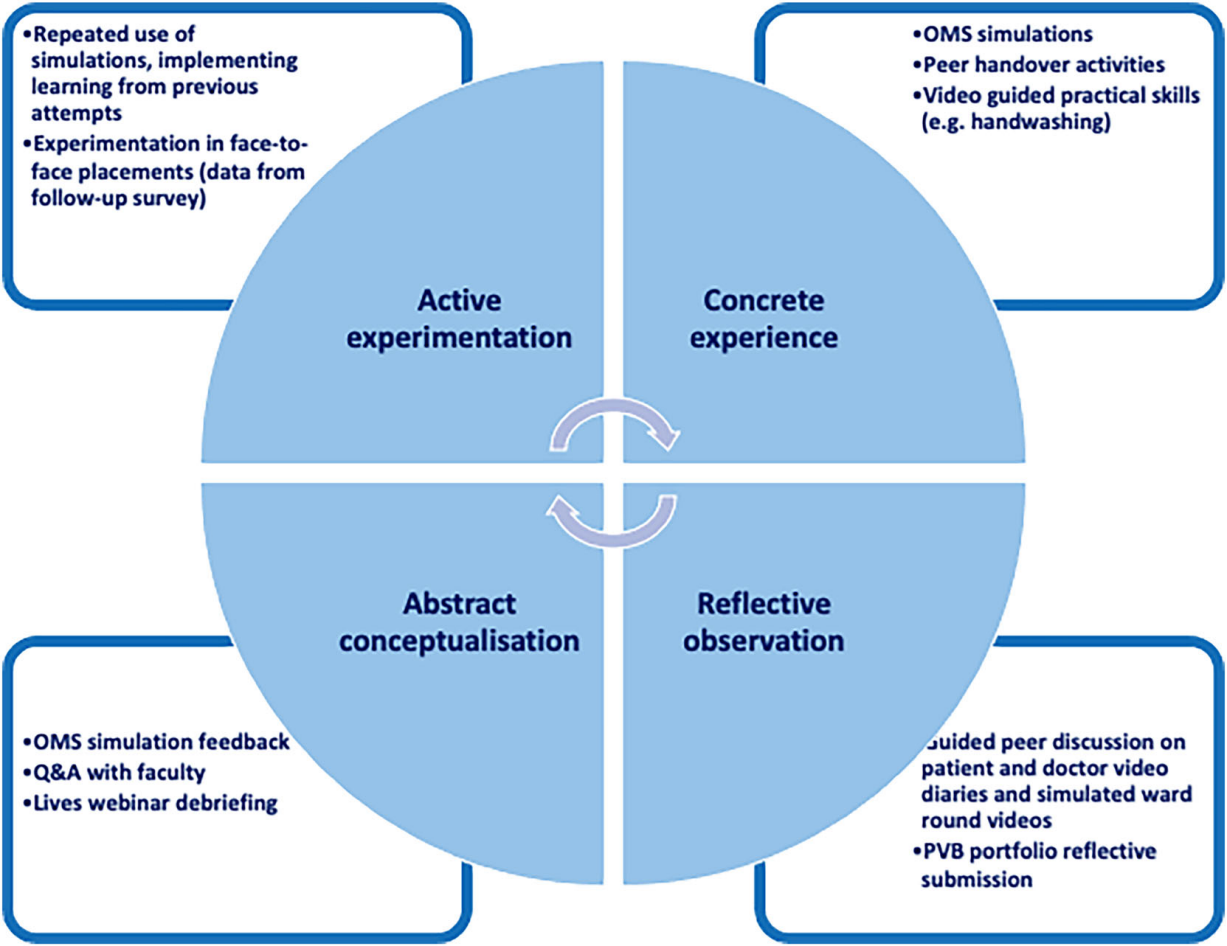
Activities mapped to Kolb's experiential learning cycle

To optimise experiential learning, we mapped activities to Kolb's experiential learning cycle^11^ as displayed in Figure 2. Students were asked to complete all self‐directed activities in pairs to maximise interactivity and maintain the collaborative spirit of experiential learning.^2^

## 2 MATERIALS AND METHODS

### 2.1 Survey design and rationale

This work was approved by the Medical Education Ethics Committee at Imperial College London (ID: MEEC1920–181). Evaluation design was informed by the New World Kirkpatrick Model.^12^ Students were invited to complete an online survey immediately after finishing the digital placement (Appendix 1). This assessed students' reaction and learning (Kirkpatrick levels 1 and 2). The same cohort was invited to complete a follow‐up survey 9 months later after their first actual face‐to‐face placement (Appendix 2). This evaluated the impact on behaviour and results (Kirkpatrick levels 3 and 4). The survey design was an iterative process, initially developed by the same eight academic teaching staff leading the course with support from a curriculum evaluation specialist (AB). Surveys included closed and open questions combining quantitative and qualitative data providing a more complete picture of the student experience.

### 2.2 Procedure

#### 2.2.1 Participation and data collection

Three hundred ten students attended the digital placement. Students were recruited to participate in evaluation using lecture shout‐outs during both digital and face‐to‐face placements. Eighty‐three respondents completed the postdigital placement survey. Twenty‐nine students completed the post‐face‐to‐face placement evaluation survey.

### 2.3 Data analysis

Anonymous data was collected. Quantitative data were analysed using a survey tool (Qualtrics) and qualitative data using a software program (Dedoose). Qualitative data were interpreted with reflexive thematic analysis as described by Braun and Clarke.^13^ The initial survey was designed to more broadly evaluate the placement therefore it did not provide the structure for preliminary analysis. The authors familiarised themselves with the data, after which excerpts of text (566 total) from the written responses were coded by NH and LW to create 102 distinct descriptive concept codes. AB completed an independent audit of concept codes. Discrepancies were identified and discussed until consensus was achieved. Through subsequent reading and analysis, these concept codes were reviewed for shared meaning and grouped into potential themes. Provisional themes were reviewed with all authors and critically assessed against our research questions, considering the extent to which they reflected the whole data set to refine final themes.

## 3 RESULTS

### 3.1 Quantitative results

At the end of the digital placement, students were asked if they felt able to perform basic tasks required for safe participation in a clinical environment (prior to the digital placement they had not received any teaching on these skills). The vast majority agreed (‘agree’ or ‘strongly agree’) that they could explain how to perform a patient assessment using an ABCDE approach (96%), calculate a National Early Warning Score (NEWS2)^14^ for patients' clinical observations (98%), explain the relationship between a NEWS2 score and likely severity of illness (93%) and escalate concerns about a patient to a senior colleague (67%), indicating high self‐perceived achievement of these goals.

At the end of the digital placement, two‐thirds of respondents agreed it had better prepared them for future placements. After completing their first actual face‐to‐face placement, 48% felt the digital placement had prepared them. Students rated digital placement as more enjoyable than traditional classroom teaching (81%); however, in the post‐face‐to‐face placement survey, the majority reported that face‐to‐face placement was both more enjoyable and more difficult (68%) than digital placement (74%). At the end of the digital placement, 83% were in favour of including digital simulations in the standard curriculum; 76% approved of continuing digital placements alongside clinical placements once regular placements resume; this dropped to 40% in the post‐face‐to‐face placement survey.

Two‐thirds of respondents agreed it had better prepared them for future placements.

### 3.2 Qualitative results

Reflexive thematic analysis of free text responses identified three superordinate themes:

1. Domain integration in digital placements helped students feel better prepared;
2. Digital experiential learning is ideally suited to early clinical learning;
3. Digital placements are a compliment, not an alternative, to face‐to‐face placements.

Representative quotations are reported in brackets after corresponding concepts; respondents' status is designated as either PD (postdigital placement survey) or PF (post‐face‐to‐face follow‐up survey).

### 3.3 Domain integration in digital placements helped students feel better prepared

There was evidence that digital placements helped students feel better prepared for future hospital placements: ‘I understand how to learn from a clinical placement in a hospital setting as a medical student and some of the key things to look out for [31PD]’ and ‘I now feel much better prepared for future hospital placements’ [13PD].

Qualitative responses suggest that the integration of domains (clinical skills, communication, professional values and behaviours) facilitated this. Some respondents identified clinical skill‐based learning as valuable and believed it would serve them well in future placements (e.g., ‘ABCDE assessment, NEWS2 score calculation … are all relevant to my future clinical placements and will be frequently used in them’ [45PD]). Communication skill exercises introduced a framework for interacting with colleagues and asking for help when needed (e.g., ‘It has given me a better idea of how to escalate concerns in an appropriate manner … how to handover/contact other team members’ [44PD]). It highlighted that ‘it is important to talk to patients to understand their experience of illness’ [13PD] and provided ‘a good structure to follow to start a patient interaction’ [49PD].

Professional values components enhanced the ‘sense of belonging’ of some students as legitimate members of the healthcare team. It helped students recognise how they ‘may be useful to the medical team’ [31PD]. This can be seen in the response, ‘It has clarified what medical students can/can't do when on clinical placements and emphasised that we do have a role to play in the team’ [27PD]). One respondent explicitly recognised ‘the fact that it was integrated helped to make sense of a placement in a different way’ [21PD]. These clinical, communication and professional skills together provide well‐rounded preparation for future placements.

Clinical, communication and professional skills together provide well‐rounded preparation for future placements.

### 3.4 Digital experiential learning is ideally suited to early clinical learning

As students progress through clinical education, they receive more responsibility and independence, which allows for experiential learning. During early clinical experiences this is (appropriately) limited to protect patient safety, with students sometimes relegated to being “observers” (e.g., ‘on the placement, I got to do very little with regard to practical work’ [15PF]. Unfortunately, this reduces learning opportunities in the early years of medical school. In contrast, in a digital environment, students can be ‘hands‐on’ with ‘no real‐life consequences’ [54PD]. Responses suggested that students valued the opportunity to explore, practice and ‘figure it out … knowing that no one else was being affected’ [49PD]. This allowed them to experience clinical decision‐making (e.g., ‘I felt like I was really in an acute care setting with my partner and we had the reigns on a case’ [52PD]) without risk. There was recognition from respondents that the digital environment facilitated this by allowing for ‘more responsibility than we normally would get at this stage of our training’ [41PD].

The digital placement also provided a safe, judgement‐free environment for students to begin exploring clinical medicine (e.g., ‘I think I felt more confident in carrying out the patient assessment, without the added pressure of having an actual patient there judging my every move’ [56PD]).

The digital placement also provided a safe, judgement‐free environment for students to begin exploring clinical medicine.

Additional advantages of the digital environment for early clinical education included the ability to navigate scenarios at an individual pace (e.g., ‘in acute situations, the doctor might not have time to stop and explain things to us … but in this simulation, there was more leeway to slow down and figure out what was happening’ [42PD]) and the opportunity to repeat scenarios as many times as needed (e.g., ‘the fact that we could repeat them as many times as we wanted, really helped’ [03PD]).

This theme highlights digital placements can integrate key elements of experiential learning in a safe, standardised, non‐judgemental environment while allowing students to learn at their own pace. This is particularly useful during early clinical education, where students are still building confidence and have fewer opportunities in hospitals.

### 3.5 Digital placements are a compliment not an alternative to face‐to‐face placements

Although digital placements are valuable (and accessible—our students were able to attend from across the world), there were clear limitations. Ultimately, digital placement activities were ‘not the real thing’. Two‐way human interaction was missing from virtual activities (e.g., ‘the simulation is great to understand the theory, but I also think that it needs a real‐life counterpart to get a feel for how patient interactions go’ [14PD]). Respondents also suggested they would have connected more easily in real interactions ‘when seeing someone physically in pain or trauma, there will be some things that will come more naturally’ [03PD]. This does not mean students did not see value in digital placements, on the contrary, it provided ‘insight into what an experience on wards with real patients might be like’ [38PD]. However, they recognised it ‘isn't a substitute for in person clinical experiences’ [38PD]. Crucially, students did not feel this was a gap that could be easily bridged with higher fidelity simulation or better resources. Though impressed with the calibre of the digital placement, they felt face‐to‐face placements are substantively different and were sceptical digital placements could truly emulate the ‘real thing’ (e.g., ‘You can't prepare someone for the hospital environment in any other way than sending them there, no matter how good your resources—it is just too chaotic’ [18PF]).

This theme highlights the importance of recognising differences between digital and face‐to‐face placements, using digital placements appropriately, and of continuing to advocate for face‐to‐face clinical exposure.

## 4 DISCUSSION

Although medical schools around the world delivered digital placements during the COVID‐19 pandemic, the majority focused on specialty‐specific knowledge and few used domain integration.^3^ In many cases, this was appropriate as digital placements were being delivered to senior students who already had substantial clinical exposure.^3^ Our work is unique as students participating had never completed a hospital placement. Early clinical experiences are particularly important for medical students' clinical reasoning, clinical communication skills and professional identity formation.^6^, ^15^ They also provide an ideal environment for experiential learning (as described by Kolb including concrete experiences, reflection and active experimentation). It is perhaps unsurprising that our results (particularly in the post‐face‐to‐face placement survey) indicate digital learning is not a real ‘substitute’ for first clinical experiences, despite grounding this in Kolb's learning theory (Figure 2) and several students comment on the lack of authenticity, which may impact deep learning.^16^ Indeed, as the pandemic progressed, medical schools have returned to face‐to‐face placements and few clinical educators would advocate for digital placements instead of traditional placements. Despite this, the digital placement was valued by students and felt to be useful, particularly as it helped prepare them for future clinical placements. Strong themes of increased confidence, preparedness and feeling they would be able to get more out of future placements emerged from our data. The unique position of our students (as first years) allows us to consider the utility of a short (1‐week) digital preparatory placement. The transition from classroom to clinical learning is recognised to provoke anxiety among medical students.^5^, ^17^ Existing literature suggests comprehensive pre‐placement preparation can assist in this transition.^18^ Our digital placement, if offered as preparatory (rather than an alternative) would represent a comprehensive pre‐placement programme, which may address this. Though requiring further curriculum space, this may be justified if it maximises placement learning and supports positive early clinical experiences, as our initial data suggest it may. Online simulations and learning experiences completed in pairs (in addition to faculty teaching) make the intervention scalable. It was delivered to over 300 students by eight faculty members, making it a viable curriculum addition. Extensive face‐to‐face simulation may well achieve the same (or better) degree of preparedness^19^, ^20^—but it would be far more costly and time‐consuming for faculty. These initial promising results justify further research to determine if digital placements do indeed have a valuable role in pre‐placement preparation.

The transition from classroom to clinical learning is recognised to provoke anxiety among medical students.

### 4.1 Future directions and limitations

The COVID‐19 pandemic forced innovation within institutions, though in the wake of this there is a temptation to return to ‘normal’. We believe medical education will profit from applying our lessons learned through the evaluation of pandemic‐era teaching and integrating them into future practice. We continue to advocate for this within our institution. The success of this initiative (along with others) supported the case for increased investment in simulation and digital learning recently approved at our institution. Though we have not yet replicated the digital placement in full, elements of the placement designed in‐house are incorporated into blended digital and face‐to‐face pre‐placement preparation.

Despite high engagement in the digital placement, evaluation survey responses were comparatively low, particularly for the post‐face‐to‐face placement survey. Respondents' views may therefore not be representative of the wider cohort due to selection bias. Unfortunately, we were unable to beta‐test our surveys due to time pressures in the context of the rapid development of teaching materials. This may have impacted survey uptake as well as potentially neglecting to capture major values or failures of the programme from the student voice (for example, if survey questions were misunderstood by student users). Though demographic data was collected for the initial survey, due to the relatively small sample size, subgroup analysis was deemed inappropriate. We used self‐reported outcomes by students (confidence and perspectives), and although valuable, this does not explore the impact of digital placements on objective student competence. Ideally, the digital placement would have been compared to pre‐placement 1‐day centralised induction. This was not possible since the digital placement was designed and delivered rapidly during the pandemic to replace cancelled placements. To adequately compare the impact on student preparedness further, an experimental investigation of digital placements compared to the current standard would be required.

## 5 CONCLUSION

Though not a substitute for in‐person clinical experiences, digital placements are a promising means of supporting students during the challenging transition from classroom to clinical learner. They provide a safe and non‐judgemental environment that approximates early clinical experiences and builds student confidence. Medical educators should consider incorporating digital placements as an accessible and feasible means to increase preparedness for clinical placements.

## CONFLICT OF INTEREST

The authors report no competing interests. The authors alone are responsible for the content and writing of this article.

## ETHICS STATEMENT

This research was approved by the Medical Education Ethics Committee (reference MEEC1920‐181) at Imperial College London.

## Supporting information


**Appendix S1.** Supporting information
