# Supplementary material for: Digital clinical placements: Student perspectives and preparedness for placements
Source: Clin Teach. 2023 Jan 4;20(1):e13558. doi: 10.1111/tct.13558 (PMC10108031; doi:10.1111/tct.13558)
Supplement: Supplementary file 1 — Appendix S1. Supporting information [file TCT-20-0-s001.docx]

## Appendix 1: initial survey immediately after completing the digital placement

Section 1: Quantitative data

1. Likert scale 1 (strongly disagree) to 5 (strongly agree). Regarding the digital simulations…
   1. Were I to observe a healthcare professional assessing a person with asthma, I feel completing this simulation would help me make sense of their thought process.
   2. I found these simulations enjoyable
   3. Seeing a virtual patient in the simulation motivated me to “get it right”
   4. I would feel bad if the patient deteriorated
   5. When clinical placements return to normal, I would still want to learn from digital simulations like this alongside clinical placements.
   6. I would feel more immersed in this simulation if it were in virtual reality (VR)
   7. Receiving a final score motivated me to repeat the simulation
   8. I am in favour of using digital simulations as part of medical education
2. Likert scale 1 (too easy) to 5 (too difficult). Regarding the digital simulations…
   1. I found the simulations (with the support given by faculty)...
3. Multiple choice. Regarding the digital simulations…
   1. In terms of doing these simulations with other students, I would most like to go through the simulations… only solo / in pairs or groups / both solo and in pairs or groups
4. Likert scale 1 (not enjoyable) to 5 (very enjoyable). Regarding the digital simulations…
   1. I found the simulations (with the support of the faculty)...
5. Multiple choice. The simulations were accessible to me - yes / no
6. Multiple choice. I had access to a Windows 10 and/or Mac system to run it myself - yes / no
7. Likert scale 1 (strongly disagree) to 5 (strongly agree). Regarding the digital placement as a whole…
   1. I can explain what ABCDE assessment is
   2. I can calculate a NEWS2 score from a set of observations
   3. I can explain the relationship between NEWS2 score and the likely severity of illness
   4. I can describe how to escalate my concerns about a deteriorating patient to a senior colleague
   5. I discussed my learning experience with my peers more than on the GP placement
   6. I now feel more prepared for future hospital placements

Section 2: Qualitative data

1. What were the elements of the digital experience that you think worked well?
2. What were the elements of the digital experience that you think could be improved and how?
3. How has the experience you had over the week influenced your understanding of your role as a medical student on a clinical placement?
4. What elements of this week’s experience do you think will be relevant to your future clinical placements?

Section 3: Demographic data

1. What is your age?
2. What gender do you identify as? (Male / Female / Prefer not to say / Other)
3. What is your ethnicity? (Multiple options / Any other ethnic group / Prefer not to say)
4. Do you identify as a widening participation student? (Yes / No / Prefer not to say)

## Appendix 2: follow-up survey after completing the face-to-face placement (nine months after the digital placement)

Section 1: Quantitative data

1. Likert scale 1 (strongly disagree) to 5 (strongly agree). The virtual hospital placement helped prepare me for my first face-to-face hospital placement.
2. Likert scale 1 (too easy) to 5 (too difficult). Compared to the face-to-face hospital placement, the digital placement was…
3. Likert scale 1 (less enjoyable) to 5 (more enjoyable). Compared to the face-to-face hospital placement, the digital placement was…
4. Likert scale 1 (strongly disagree) to 5 (strongly agree). Now that placements have returned to normal, I would still like to learn from digital placements alongside face-to-face placements.

Section 2: Qualitative data

1. What challenges did you face during the face-to-face hospital placement you have just experienced? Did elements of the digital hospital placement influence the way you approached these? How?
2. What elements of the clinical skills teaching during the digital hospital placement were most relevant and what helped prepared you the most for your face-to-face placement? Why? (Clinical skills teaching in the digital placement included the OMS simulations, elearning on understanding observations and NEWS2, and webinars discussing patient management in the OMS simulations .)
3. What elements of the professional values and behaviour teaching during the digital hospital placement were most relevant and what helped prepare you the most for your face-to-face placement? Why? (Professional values and behaviour teaching in the digital placement included elearning and webinars on navigating the hospital system and understanding patients experiences of illness.)
4. What elements of the clinical communications teaching during the digital hospital placement were most relevant and what helped prepare you the most for your face-to-face placement? Why? (Clinical communications teaching included elearning and webinars on handovers and calling for help as well as teamwork and appropriate patient interactions.)
5. What other teaching would have been useful during the digital hospital placement to help you prepare for the actual hospital placement?
